# Supplementary material for: Predictors of mortality in treatment experienced HIV-infected patients in northern Tanzania
Source: PLoS One. 2020 Oct 8;15(10):e0240293. doi: 10.1371/journal.pone.0240293 (PMC7544106; doi:10.1371/journal.pone.0240293)
Supplement: Attachment — Submitted filename: Response to Reviewers.docx [file pone.0240293.s001.docx]

**Reviewer 1**

1. **Comment:** Introduction – Need to better justify why early and late mortality factors may differ (i.e., justify the need for this paper).

**Response:** The factors associated with early mortality have been well-described while data regarding those associated with late mortality are limited (Please see response to comment #3 also). One objective of this analysis was to better understand the factors which may be associated with long-term mortality.

To further emphasize the value of this study, we added additional content to the introduction (Please see changes (lines 62-64) in the introduction). Notably, mortality among people living with HIV have improved in low income settings but remain lower than background populations. Thus, outcomes among PLWH engaged in care could be further optimized.

2. **Comment:** Introduction – Wouldn’t late presentation to care cause long-lasting damage to the immune system?

**Response:** Yes. The available evidence suggests that late presentation to care is associated with immunologic activation and inflammation that persist despite antiretroviral therapy (ART). This may be an important contributor to the increased non-AIDS defining mortality among PLWH on ART in comparisons to HIV-uninfected populations. It remains unclear how to measure this immunologic abnormality in patients beyond evaluating the extent of their HIV disease prior to ART initiation. In our cohort, nadir CD4 count was not predictive of mortality.

3. **Comment:** Introduction – Are you suggesting that the ARVs or new risk factors (such as behavioral ones like alcohol, smoking) would be late but not early mortality RFs?

**Response:** The existing literature suggest that the overall mortality among persons living with HIV (PLWH) initiating ART in sub-Saharan Africa (SSA) is driven primarily by early deaths associated with AIDS, which are a consequence of late ART initiation. This early mortality is a continuation of the high mortality seen in PLWH prior to the initiation of ART. For this reason, factors that influence early mortality are largely HIV-related clinical factors and reflective of advanced disease (i.e. low CD4 count). Certainly, behavioral and psychosocial risk factors may play a role but late presentation to care remains the major factor challenging the efficacy of ART in SSA at the population level.

4. **Comment:** Methods – Explain what ‘established infection receiving care’ means. Was there a minimum # years?

**Response:** Please see changes made in lines 86-90 for additional clarification. All patients with ‘established infection receiving care’ were followed at one of two HIV clinics but not all were on ART. This analysis was limited only to those on ART.

5. **Comment:** Methods – A bit more details on the random point system – not familiar with it and how it can be implemented in Tanzania; what was the point of the sampling approach? To get a sample that represents the entire district? Or all of Tanzania? All of Africa?

**Response:** A random time point sampling approach was utilized because a limited number of patients at each clinic could be enrolled per day due to staffing limitations and interview length. Please see changes made in lines 103-106 of the methods which provides this clarification. Also, please refer to citation 15 for additional details regarding the sampling at these two HIV clinics.

6. **Comment:** Methods – Prefer the term sex to gender, although am aware that transgender people would not self-identify in Tanzania’s current situation

**Response:** All references to gender were changed to sex in the manuscript.

7. **Comment:** Methods – Was PHQ-9 done at every visit (every 6 months)?

**Response:** The PHQ-9 was repeated at every visit (every 6 months). Only the baseline PHQ-9 (performed at the first visit) is included in this analysis.

8. **Comment:** Methods – No mention of indications to start ART during the study.

**Response:** We did not collect indications for starting ART in these patients. Tanzanian HIV treatment guidelines have largely followed treatment guidelines set by the WHO. Current WHO treatment guidelines support a “treat all” approach. However, guidelines prior to 2016 supported a selective treatment approach based on clinical severity of HIV disease and/or CD4 count. For this reason, many of our participants had advanced HIV disease around the time of diagnosis.

9. **Comment:** Methods – Why not use VL <1000 as evidence of adherence? Self-report is notoriously not good.

**Response:** We agree that viral load is a better measure of adherence. However, viral load likely mediates the relationship between predictor variables and mortality. For this reason, we excluded viral load from the regression analysis. We attempted to collect accurate adherence information by asking two adherence questions in two different ways, including a visual analog scale. If a participant indicated that they were not adherent to at least one of these two questions, they were categorized as not adherent.

10. **Comment:** Methods – No discussion of loss to follow-up or transfers out in the cohort and how it was mitigated, ascertained, etc.

**Response:** We’ve added a loss to follow-up sub-section in the methods. Please see lines 138-144.

11. **Comment:** Methods – Not including the HIV-uninfected cohort (to comment on their mortality rates) seems like missed opportunity.

**Response:** We agree that the inclusion of the mortality rate among the HIV-uninfected cohort could have added additional context to the mortality rate described among this ART experienced cohort. However, we decided not to include the HIV-uninfected cohort because in the analysis, the number of participants who died in that cohort was very small. These very few deaths would have allowed very limited comparison between the survival curve of the HIV-uninfected cohort and that of those who were on ART treatment because estimates would need to be unadjusted for key covariates. Exploring causes and rates of mortality among the HIV-uninfected adults was beyond the scope of this analysis.

12. **Comment:** Methods – How did you handle time varying predictors? Or were all predictors at the entry to cohort.

**Response:** All predictor variables were taken from the baseline survey at enrollment.

13. **Comment:** Methods – Justify why you did these 3 models.

**Response:** We elected to use a block-wise inclusion of *a priori* selected variables. The independent variables of model 1 consisted of relevant sociodemographic variables, model 2 included of relevant clinical variables (controlled for age and gender), and model 3 was a combined model. We believe this approach was more in line with an *a priori* theory driven approach to model building. Please see lines 168-171 for additional clarification in the manuscript.

14. **Comment:** Methods – No attempt made to adjust for the fact that patients who were retained for 700 days on ART and entered the study may be different from the overall clinic population and/or PLWH in the area. Nor was this major limitation well-acknowledged

**Response**: We agree that this treatment experienced cohort is not representative of the background clinic cohort or PLWH in this region. This was listed as a limitation (please see lines 339-344) in the discussion.

15. **Comment:** Methods – No mention of TB throughout this paper, the 1st cause of death in PLWH in SSA.

**Response:** While the clinical syndromes among a proportion of the decedents in this study may be consistent with tuberculosis, we were unable to reliably attribute tuberculosis as a cause of death due to the lack of relevant diagnostic information.

16. **Comment:** Methods – They did not refer (or failed to mention it) depressed patients for mental health care, which raises some possible ethical concerns. Did you assess suicidal thoughts in persons reporting depression? Did you have a safety plan or mental health expert involved?

**Response:** The PHQ-9 has one item which evaluates suicidal thoughts and this item evaluates if the patient/participant has “Thoughts that you would be better off dead, or of hurting yourself in some way? We did not evaluate this item separately as we scored all items of the PHQ-9 combined.

We believe that the findings of this current analysis as well as previous publications from this cohort (please see citations 11, 15, and 16) highlights the need to increase the availability of mental health care among PLWH in SSA. Unfortunately, the number of mental health providers in Tanzania remains low and this was certainly true from 2008 through 2012, the time period of this study.

17. **Comment:** Results – Many were excluded as not on ART; why and didn’t this introduce a strong bias into your analysis?

**Response:** We report this bias in our discussion section (Please see lines 334-337 and the response to comment #14). However, the objective of this analysis was to understand mortality outcomes among those on ART. Overall, we demonstrated that mortality was low among this group and these results highlight the potential value in earlier diagnosis and treatment as well as further expansion of HIV treatment (Please see lines 245-250). However, our results indicate that even among those engaged in treatment, not all are realizing the full benefits of ART.

18. **Comment:** Results – These are very old data (from 2008); does it diminish current impact?

**Response:** While data collected for this study finished in 2012, we believe these data have current value because ART access continues to expand throughout SSA. It may be more relevant than ever to understand patient-level or facility-level characteristics that predict poor outcomes among those on ART and the consequences of delays in linkage to care.

19. **Comment:** Results – Make it clear what the average time on ART was at censoring.

**Response:** The time on ART was not censored but rather the median (IQR) time on ART in years from the date each participant started ART to their baseline survey date. For each study participant, we collected the start date of ART and the date of enrollment. The time on ART was determined by subtracting these two values.

**7Comment:** Results – Give more details on how mortality was determined. What was time from death to when the clinic became aware? Did you ever have inpatient records? How often was nothing other than outpatient file available? How often was verbal autopsy performed and with whom? How often did reviewers disagree, etc.

**Response:** We did not collect data regarding the time from participant’s death to the time that the clinic was aware of the death. We did collect the time of death to the best of the clinic’s and the study team’s knowledge. Regarding the verbal autopsy procedure, we used death certificates, inpatients records, laboratory records, and available HIV clinic documents if available. The methods section (please see lines 149-151) has been appropriately updated. Disagreement between the reviewers is reported in lines 199-200 of the results.

20. **Comment:** Results – Table 2, why not use HIV VL as adherence, since having repeated HIV VL is one of the unique elements of this cohort?

**Response:** Please see the response to comment #9.

21. **Comment:** Results – Table 3 not very helpful.

**Response:** We included Table 3 to show the lower proportion of virologic suppression among decedents in comparison to survivors (related to comments #9 and 21). In addition, please see our response to #24. Table 3 could be moved to a supplemental appendix.

22. **Comment:** Discussion – Must acknowledge the limitations of verbal autopsy

**Response:** We added the limitations of verbal autopsy to the discussion. Please see lines 342-344.

23. **Comment:** Discussion – Do you think most deaths are due to HIV/AIDS-related causes in a virally suppressed, high CD4 cohort? If so, why? Which HIV/AIDS-related causes of deaths are emerging or persisting here?

**Response:** The majority of our deaths occurred within the first 1-2 years of enrollment. While the proportion virally suppressed was high in the cohort, viral load suppression among decedents appears lower in comparison to survivors. For this reason, we believe the verbal autopsy findings are supported by viral load data. Please see lines 258-261 for additional clarification.

24. **Comment:** Discussion – If you wish to highlight depression in this cohort, why not demonstrate that depression led to reduced viral suppression and reduced CD4, which led to increased mortality. Isn’t that the mechanism. Or suicide – was it ascertained at all in depressed people

**Response:** A previous study in this cohort found that depression led to reduce ART adherence and viral suppression. Please refer to lines 295-297 and citation 16.

**Reviewer 2**

1. **Comment:** Title page – A symbol (e.g. asterix) for corresponding author should be added on the corresponding author’s name. In addition, authors may consider adding key words.

**Response:** An asterisk was added to the corresponding author (please see line 4). In addition, we added key words (please see line 16).

2. **Comment:** Methods – Line 87, page 5 reads “Any patient 18 – 65 years old and residing in the districts of Moshi urban or Hai of the Kilimanjaro region…” It might be enlightening to explain the justification for the upper age limit.

**Response:** At the time of the study, most adult HIV statistics were limited to ages 18-49. In anticipation of ART related survival gains we expanded study eligibility to ages 18-65.

3. **Comment:** Methods – Given the established role of socioeconomic levels as a determinant of mortality, authors may consider adding to this analysis variables such as employment status or estimate for monthly income which explain better socioeconomic levels. As authors have correctly pointed out in the discussion, ‘level of education’ could be a poor measure of socioeconomic levels and therefore leading to ‘unexpected’ results.

**Response:** We agree with the reviewer. In subsequent survey rounds, we were able to calculate a socioeconomic score based on ownership of household items. However, these questions were not asked in the baseline interview. The baseline interview was used to generate baseline variables. Because a large number of deaths occurred within the first few study rounds, the use of the socioeconomic score generated from variables in later rounds would lead to missing values; dropping these observations would lead to bias.

4. **Comment:** Similarly, given accumulating data pointing to the contribution of non-communicable diseases (hypertension and diabetes) to mortality among HIV patients on long treatment to ART, authors may consider adding to this analysis, variables such as BMI, blood pressure, blood glucose, liver and kidney function test. Also, it is important as part of discussion for this manuscript to build a case for routine HIV care and treatment to include some of these measures as well as conducting periodic screening for hypertension and diabetes as a strategy for reducing mortality in HIV patients who have been on long term exposure to ART.

**Response:** We agree that deaths attributed to non-communicable diseases are likely to increase over time among persons living with HIV with treatment-experience as suggested in recent data. However, the number of deaths limited the number of variables that could be explored in the regression analysis, and HIV/AIDS was the most common cause of mortality in this cohort.

5. **Comment:** Analysis: Line 139, page 7 reads “In multivariate analysis, we constructed three models…” It was not made clear on the justification of this analysis plan. Authors may consider elaborating this.

**Response:** We elected to use a block-wise inclusion of *a priori* selected variables. The independent variables of model 1 consisted of relevant sociodemographic variables, model 2 included of relevant clinical variables (controlled for age and gender), and model 3 was a combined model. We believe this approach was more in line with an *a priori* theory driven approach to model building. Please see lines 168-171 for additional clarification in the manuscript.

6. **Comment:** page 17 first paragraph reads “Patients receiving care at the CTC in regional referral hospital in comparison to the larger zonal referral hospital were found to have higher risk of death”. While I completely agree with the author’s arguments. Authors may consider adding to their argument a potential indirect influence of socioeconomic status particularly on the side of patients given that patients’ socio-economic level will determine whether or not they choose to be cared at a public facility.

**Response:** We agree with the reviewer. The observed difference in mortality between these two facilities could be confounded by another factor, particularly socioeconomic status. Please see the changes in lines 307-311.
