# Supplementary material for: Predictors of mortality in treatment experienced HIV-infected patients in northern Tanzania
Source: PLoS One. 2020 Oct 8;15(10):e0240293. doi: 10.1371/journal.pone.0240293 (PMC7544106; doi:10.1371/journal.pone.0240293)
Supplement: Attachment — Submitted filename: Moshi_HIV_ comments.docx [file pone.0240293.s002.docx]

Methods

82 Participants

Whats was the age of CHAT patients and from what regions? Please include that

Consider this revision: This study included patients 18-65 years old and residing in the districts of Moshi Urban or Hai of the Kilimanjaro Region, and with plans to stay in the region for the foreseeable future, were eligible for enrollment. The enrolled patients had known HIV infection diagnosis and receiving care at the HIV clinics of the Kilimanjaro Christian Medical Centre (KCMC) or Mawenzi Regional Referral Hospital (MRRH) which are private zonal referral hospital and public regional hospital respectively.

Consider adding a map of Tanzania showing the regions which KCMC serves. In addition, you can have a zoom out map of Kilimanjaro region or Moshi municipality showing relative locations of Mawenzi and KCMC

104: Please describe the random time point concisely to enhance understanding of readers

144 describe the verbal autopsy system and questionnaire used and provide references

Consider adding participants flow chart showing both survivors and decedents and various outcomes to ensure the readers follow up various analyses

175 should be age and sex descriptive and then median days of enrollment should follow

179 Table 1 please re-organise: include age categories, remove the unadjusted HR and append HIV RNA results from table 3 and to make table 1. Consider presenting categories of time to ART and nadir CD4. Median values may be included in results as text. Consider adding missing value and removing denominators in each cell. Streamline the tables to simplify visualisation and reference. Revise the title of table. Suggestion. Distribution of demographic characteristics, clinical and laboratory findings by mortality status among a cohort of HIV-infected patients on antiretroviral therapy in northern Tanzania, 2008-2012

204 add a column of unadjusted HR from table one before the column for model 1 to easy readers visualisation of comparison. Cells with missing data should be described.

231 Discussion

Have a paragraph to discuss your cohort using table one variable, at minimum age and sex distribution and mortality status the discuss mortality rate to improve the flow.

CD4 counts, HIV RNA and sexual differences in baseline characteristics can be discussed in the context of Tanzania HIV Impact Survey (THIS 2016-2017) a population based household national representative survey with regional results. <http://library.tacaids.go.tz/bitstream/handle/123456789/135/THIS%202016%202017_Final%20Report__1.08.19_TS-1.pdf?sequence=1&isAllowed=y>

242 other parts of SSA especially southern African and northern Moshi may have different subtypes which may have different clinical course and hence difference in mortality. (<https://pubmed.ncbi.nlm.nih.gov/23118882/>). You may also want to look at host and HIV variation seroconverting in (<https://pubmed.ncbi.nlm.nih.gov/25473042/>)

More discussion is warranted on the association of high education and mortality

251 consider revision of the term judged
