# Supplementary material for: Predictors of mortality in treatment experienced HIV-infected patients in northern Tanzania
Source: PLoS One. 2020 Oct 8;15(10):e0240293. doi: 10.1371/journal.pone.0240293 (PMC7544106; doi:10.1371/journal.pone.0240293)
Supplement: Attachment — Submitted filename: Response to Reviewers.docx [file pone.0240293.s003.docx]

**Reviewer 1**

**Comment:** This paper analyzes mortality predictors among persons with HIV in Northern Tanzania using data from 2008-2012, a period that one could considered the 'early ART era' there (with mostly D4T-based regimens), making the results less relevant to now. The analysis suffers from multiple issues that cannot be completely addressed although the authors have thoughtfully responded to my comments. The fact that the data were harmonized from multiple diverse cohorts and multiple forms of selection bias in the patient characteristics cannot really be addressed also reduce the impact of these results. Repeated measures of depression and inability to leverage an HIV-negative cohort (too demographically different) are missed opportunities. It is my opinion that this does not sufficiently build HIV-related knowledge to warrant publication in Plos One.

**Response:** We thank the reviewer for this comment. ART regimens change over time, both in global and domestic settings. Since 2006 in the United States and since the advent of their widespread use in Tanzania, ART has affected a very strong influence on mortality. Exactly how the differences in regimens from then and now might affect outcomes in this analysis are unclear, but we believe they would be negligible as we are not aware of applicable data to suggest mortality differences between regimens then and now.

We have clarified the contribution of the various cohorts recruited into the CHAT study and which ones contributed to this analysis (please see lines 86-87). We acknowledge selection bias in this study and it is reported as a limitation. All other concerns of the reviewer were previously addressed.

**Reviewer 3**

**METHODS**

1. **Comment**

82 Participants

Whats was the age of CHAT patients and from what regions? Please include that.

Consider this revision: This study included patients 18-65 years old and residing in the districts of Moshi Urban or Hai of the Kilimanjaro Region, and with plans to stay in the region for the foreseeable future, were eligible for enrollment. The enrolled patients had known HIV infection diagnosis and receiving care at the HIV clinics of the Kilimanjaro Christian Medical Centre (KCMC) or Mawenzi Regional Referral Hospital (MRRH) which are private zonal referral hospital and public regional hospital respectively.

Consider adding a map of Tanzania showing the regions which KCMC serves. In addition, you can have a zoom out map of Kilimanjaro region or Moshi municipality showing relative locations of Mawenzi and KCMC

**Response:** Please see changes to the methods section (lines 86-87). First, we have attempted to clarify the different cohorts to make it easier for the reader to understand that we focused this analysis on the clinical cohort, which is comprised of patients who were enrolled from the two HIV clinics in Moshi. We then use lines 92-97 to provide further information about this cohort. Second, please see lines 106-109 for details regarding age criteria and region of residence of eligible participants. Third, please see Figure 1 for a map of the Kilimanjaro Region with the regions served by KCMC. In addition, we have labeled KCMC and MRRH.

2. **Comment:** Please describe the random time point concisely to enhance understanding of readers.

**Response:** Please see lines 114-124 for additional details regarding the random time point system.

3. **Comment:** Describe the verbal autopsy system and questionnaire used and provide references.

**Response:** Please see lines 163-164. The verbal autopsy tool used in this study was adapted from the World Health Organization Verbal Autopsy Standard.

4. **Comment:** Consider adding participants flow chart showing both survivors and decedents and various outcomes to ensure the readers follow up various analyses

**Response:** Please see Figure 2 which provides participant enrollment flow and outcomes.

**RESULTS**

1. **Comment:** Should be age and sex descriptive and then median days of enrollment should follow.

**Response:** Please see changes made to this section in lines 196-199.

2. **Comment:** Table 1 please re-organize: include age categories, remove the unadjusted HR and append HIV RNA results from table 3 and to make table 1. Consider presenting categories of time to ART and nadir CD4. Median values may be included in results as text. Consider adding missing value and removing denominators in each cell. Streamline the tables to simplify visualization and reference. Revise the title of table. Suggestion. Distribution of demographic characteristics, clinical and laboratory findings by mortality status among a cohort of HIV-infected patients on antiretroviral therapy in northern Tanzania, 2008-2012

**Response:** Please see the changes to Table 1. First, we have included categorical variables for age, time on ART, and nadir CD4. Second, unadjusted HR were removed. Third, we appended the HIV RNA results from Table 3 and removed Table 3 from the document. Fourth, we have revised the title based on the reviewer’s suggestion. Finally, we have added missing values for variables with missing data.

3. **Comment:** Add a column of unadjusted HR from table one before the column for model 1 to easy readers visualization of comparison. Cells with missing data should be described.

**Response:** The unadjusted HR from Table 1 are moved to Table 2. In Table 2, a hash (-) is now added to indicate missing cells. To further help the reader understand the 3 models, we have a description of the models at the foot of the table.

**Discussion**

1. **Comment:** Have a paragraph to discuss your cohort using table one variable, at minimum age and sex distribution and mortality status the discuss mortality rate to improve the flow.

**Response:** Please see changes in the second paragraph of the discussion (lines 293-304-297).

2. **Comment:** CD4 counts, HIV RNA and sexual differences in baseline characteristics can be discussed in the context of Tanzania HIV Impact Survey (THIS 2016-2017) a population-based household national representative survey with regional results. <http://library.tacaids.go.tz/bitstream/handle/123456789/135/THIS%202016%202017_Final%20Report__1.08.19_TS-1.pdf?sequence=1&isAllowed=y>

**Response:** The differences in the proportion of men and women enrolled in our study reflects known differences in HIV prevalence between men and women in Tanzania. We have stated this in the document and cited THIS 2016-2017 (citation 21). Furthermore, the proportion of participants virally suppressed is similar to that reported in the THIS 2016-2017 study. However, regional viral load data for Kilimanjaro in THIS 2016-2017 were limited because of a low sample size so we could not make further comparisons between our findings and that reported for Kilimanjaro in THIS.

3. **Comment:** Other parts of SSA especially southern African and northern Moshi may have different subtypes which may have different clinical course and hence difference in mortality. (<https://pubmed.ncbi.nlm.nih.gov/23118882/>). You may also want to look at host and HIV variation seroconverting in (<https://pubmed.ncbi.nlm.nih.gov/25473042/>)

**Response:** This is an important topic and may be an explanation for the differences in mortality seen in our study compared to prior reports from other countries in SSA. Unfortunately, our data limits further exploration of this topic.

4. **Comment:** More discussion is warranted on the association of high education and mortality

**Response:** We do not have a clear explanation for the association between high education and mortality. The existing evidence suggest that HIV mortality decreases with increasing education attainment. One explanation we have suggested is that education is not an ideal measure of socioeconomic status in some settings. We have listed reasons for this in the discussion (lines 352-358). Measurement of socioeconomic status is best measured reducing components of individual, household, and community resources into one variable.

5. **Comment:** Consider revision of the term judged

**Response:** The term “judged” was changed to “adjudicated”.
