# Supplementary material for: Predictors of mortality in treatment experienced HIV-infected patients in northern Tanzania
Source: PLoS One. 2020 Oct 8;15(10):e0240293. doi: 10.1371/journal.pone.0240293 (PMC7544106; doi:10.1371/journal.pone.0240293)
Supplement: Attachment — Submitted filename: Response to Reviewers.docx [file pone.0240293.s004.docx]

**Reviewer 2**

**Comment:** For a manuscript on mortality among HIV infected patients on ART, it would be unfair to pass without a mention of high Body Mass Index, hypertension and type 2 diabetes mellitus (high Blood Glucose) as potential risk factors to mortality. If such information were not collected, authors should strongly consider to include these as part of their study limitations.

**Response:** We agree with the reviewer that noncommunicable diseases and their risk factors are relevant predictors of long-term mortality among persons living with HIV. Because this is missing from our analysis, we have included this as a study limitation. Please see lines 331-335.
